# Integration of Antimicrobials and Delivery Systems: Synergistic Antibiofilm Activity with Biodegradable Nanoemulsions Incorporating Pseudopyronine Analogs

**DOI:** 10.3390/antibiotics12081240

**Published:** 2023-07-28

**Authors:** Jungmi Park, Neel Mahida, Gabrielle Ho, Elizabeth Pena, Jessa Marie V. Makabenta, Stanley Aneke, Mingdi Jiang, Leah M. Bouthillette, Stephanie E. Holz, Muhammad Aamir Hassan, Amanda L. Wolfe, Vincent M. Rotello

**Affiliations:** 1 Department of Chemistry, University of Massachusetts Amherst, Amherst, MA 01003, USA; jungmipark@umass.edu (J.P.); nmahida@umass.edu (N.M.); gho@umass.edu (G.H.); epena@umass.edu (E.P.); jmakabenta@umass.edu (J.M.V.M.); saneke@umass.edu (S.A.) mingdijiang@umass.edu (M.J.); muhammadaami@umass.edu (M.A.H.); 2 Department of Chemistry and Biochemistry, University of North Carolina Asheville, Asheville, NC 28804, USA; lbouthil@ucsc.edu (L.M.B.); sholz@unca.edu (S.E.H.); awolfe@unca.edu (A.L.W.)

**Keywords:** biofilm infections, nanoemulsions, pseudopyronine analogs, essential oils

## Abstract

Multi-drug-resistant (MDR) bacteria, including methicillin-resistant *Staphylococcus aureus* (MRSA), pose a significant challenge in healthcare settings. Small molecule antimicrobials (SMAs) such as α-pyrones have shown promise as alternative treatments for MDR infections. However, the hydrophobic nature of many SMAs limits their solubility and efficacy in complex biological environments. In this study, we encapsulated pseudopyronine analogs (PAs) in biodegradable polymer nanoemulsions (BNEs) for efficient eradication of biofilms. We evaluated a series of PAs with varied alkyl chain lengths and examined their antimicrobial activity against Gram-positive pathogens (*S. aureus*, MRSA, and *B. subtilis*). The selected PA with the most potent antibiofilm activity was incorporated into BNEs for enhanced solubility and penetration into the EPS matrix (PA-BNEs). The antimicrobial efficacy of PA-BNEs was assessed against biofilms of Gram-positive strains. The BNEs facilitated the solubilization and effective delivery of the PA deep into the biofilm matrix, addressing the limitations of hydrophobic SMAs. Our findings demonstrated that the PA2 exhibited synergistic antibiofilm activity when it was loaded into nanoemulsions. This study presents a promising platform for addressing MDR infections by combining pseudopyronine analogs with antimicrobial biodegradable nanoemulsions, overcoming challenges associated with treating biofilm infections.

## 1. Introduction

Multi-drug-resistant (MDR) bacteria annually cause at least 2 million infections, leading to 23,000 deaths and increased hospitalizations each year [1]. Some of the most concerning MDR bacteria include methicillin-resistant *Staphylococcus aureus* (MRSA) [2]. MDR bacteria are challenging to treat due to their adapted ability to tolerate high levels of therapeutics [3,4,5]. Not addressing MDR bacterial infections contributes to increased persistence of infection, even resulting in mortality [6]. Biofilms formed by MDR bacteria are particularly challenging, not only from the inherent ability of associated bacteria to tolerate high levels of therapeutics [7], but also the protective environment, resulting in antibiotic therapy being ineffective [8]. The extracellular polymeric substances (EPSs) matrix in a biofilm, in particular, is a barrier to most antibiotics, resulting in increased antimicrobial resistance and prolonged infection [9].

**Figure 1:**
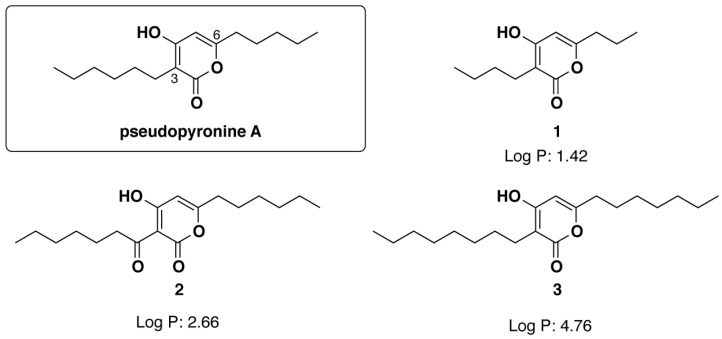
Chemical structures of pseudopyronine A and pseudopyronine analogs (PA) 1, 2, and 3.

Small molecule antimicrobials (SMAs) provide effective therapeutics and can offer a large design space that can be explored for drug development as an alternative solution to MDR infections [10]. α-Pyrones are a class of natural products produced by a variety of microorganisms as both biosynthetic precursors and secondary metabolites that have been found to possess antibiotic, antifungal, cytotoxic, and anti-atherosclerotic activity [11,12,13,14,15]. Pseudopyronine A (Figure 1) and pseudopyronine B are α-pyrone natural products produced by multiple species of bacteria that have saturated alkyl chains on C3 and C6 [16,17,18,19]. The pseudopyronines act as antibiotics against both resistant and non-resistant Gram-positive bacteria via selective membrane disruption and inhibition of fatty-acid synthase (FAS) II [19,20,21]. Recently, we synthesized and evaluated a series of C3/C6 pseudopyronine analogs and found that alkyl chain length at these positions directly affects antibacterial activity with analogs with longer alkyl chains (up to 6/7 carbons) showing the highest potency against susceptible strains of *S. aureus* and *B. subtills* both Gram-positive pathogens [22]. 

Hydrophobic SMAs have limited aqueous solubility, making them challenging to use in complex biological environments like biofilms [23]. Combination strategies employing dual antimicrobials to combat biofilm infections have been studied; however, their efficacy toward mature biofilms is limited due to heterogeneous compositions deactivating many therapeutics [24]. 

Nanoemulsions involving essential oils have shown promising potential in treating bacterial infections with broad-spectrum activity, high biocompatibility, and a high barrier against resistance development [25,26]. Polymeric carriers based on poly(oxanorbornenimide) (PONI) backbones have been utilized for antimicrobial delivery [27]. In our previous studies, we introduced functionalized PONI-polymers incorporating guanidinium, maleimide, and tetramethylene glycol monoethyl ether (PONI-GMT) to create nanoemulsions with nature-derived essential oils including eugenol, carvacrol, linalool, and methyl eugenol [26]. We further explored this system to load hydrophobic antimicrobial therapeutics for the synergistic treatment of bacterial biofilms, leveraging the amphiphilic properties of nanoemulsions. Therefore, we hypothesized that polymer-based biodegradable nanoemulsions (BNEs) can be utilized along with pseudopyronine analogs (PAs) to encapsulate and solubilize to reach deep into the EPS matrix and produce enhanced antibiofilm activity. For this study, we chose to examine three compounds with varied activity from the original series, PA1 (C = 3,4), PA2 (C = 6,7), and PA3 (C = 7,8) [28]. From these leads, PA2 had the best synergistic antibiofilm activity with the BNE. PA-BNE reduced bacterial viability against *S. aureus* and MRSA and *B. subtilis* biofilms compared with when PA2 worked alone. Notably, PA-BNEs showed minimum cytotoxicity to fibroblast cells. Taken together, these studies demonstrated that careful choice of therapeutic and carrier presents a strategy to access a large design space of small molecules to address limitations of SMAs including solubility and biofilm penetration.

## 2. Results

### 2.1. Antimicrobial Activity of Analog Compounds against Gram-Positive Planktonic Bacteria

The intrinsic antimicrobial activity of analogs was evaluated against Gram-positive bacteria including MRSA by determining minimum inhibitor concentration (MIC) values (Table 1). PA1 was found to have MIC of 250 mg/L against *S. aureus* and *B. subtilis*, respectively. The most potent activity against *S. aureus* was observed for PA2 with MIC value of 0.5 mg/L. For longer alkyl chain analogs, PA2 and 3 showed more potent activity against *S. aureus* with 0.5 mg/L and 25 mg/L, respectively. We also tested the analogs against a drug-resistant strain of *S. aureus*, specifically MRSA, where the MIC values were higher than *S. aureus*.

### 2.2. Fabrication of PAs-Loaded Biodegradable Nanoemulsions (PA-BNEs)

#### 2.2.1. Solubility of PAs in Eugenol

BNEs were fabricated by emulsifying essential oils (eugenol) with amphiphilic polymers using an oil–water interface and cross-linking [26]. The essential oil can be used to load hydrophobic components [29]. In this study, eugenol was chosen for its wide spectrum of antimicrobial activity and ability to disrupt biofilm when it was delivered with BNEs. PAs were dissolved in eugenol, and the solubility of each compound was recorded (Table 2). The solubility was high for PAs close to log P of eugenol (log P: 2.49). The PA2 have similar log P values of eugenol, affording the highest concentrations of around 100 mg/mL (PA2 log P: 2.66). However, PA1 and PA3 have higher or lower log P values (PA1 log P: 1.42, PA3 log P: 4.76), and show somewhat lower solubility compared with PA2. Taken together, PA2 was chosen for further studies due to its higher antimicrobial activity and better carrier solubility.

#### 2.2.2. Generation and Characterization of PA-BNEs

**Figure 2:**
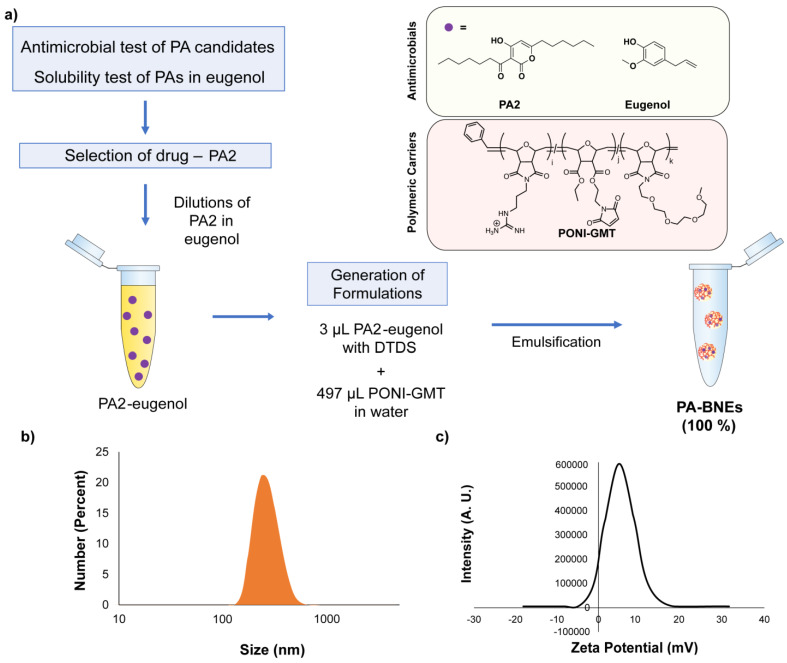
(**a**) Schematic representation of the workflow for preparation of PA-loaded nanoemulsions (PA-BNE) after solubility test and antimicrobial testing; (**b**) hydrodynamic size of PA-BNE measured by dynamic light scattering (DLS); (**c**) intensity graph of zeta-potential measurement.

Eugenol was used to dissolve PA2 (up to 24 mg/mL), and serial diluted to generate different formulations of PA2-loaded nanoemulsions (PA-BNE) (Figure 2a). In each fabrication, 500 µL of PA-BNE solution containing different PA2 levels was afforded and defined as 100% solution (*v/v*). The hydrodynamic size of PA-BNEs was measured by dynamic light scattering (DLS) and formed around 290 nm with narrow size distributions (polydispersity index: 0.08) (Figure 2b, Figure S1). The measured zeta potential of PA-BNEs was +10 mV, attributed to positively charged guanidinium groups on the polymers (Figure 2c).

### 2.3. Antimicrobial Activity of PA-BNE 

#### 2.3.1. Antimicrobial Activity of PA-BNEs

We next evaluated the antimicrobial activity of PA-BNE against MRSA (IDRL-6169). The minimum bactericidal concentrations (MBCs) were determined for each material PA2 and BNE. The PA2 alone was 12 mg/L and BNE was 16% (Table 3). Then, we varied PA2 loading to generate PA-BNEs and check the MBC. We observed 8% concentrations of BNE with 4 mg/L, showing eradication of MRSA the next day. The MBCs of PA2 were a third time less with BNE, and the MBC of BNE was half-fold than when treated alone, indicating a positive additive effect of those two systems.

#### 2.3.2. Antibiofilm Activity of PA-BNEs

**Figure 3:**
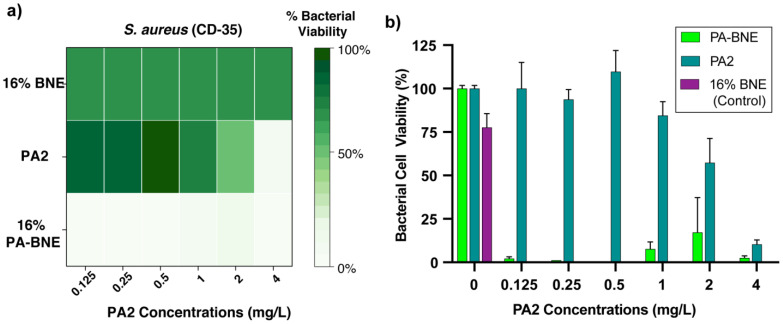
(**a**) Minimum biofilm bactericidal concentrations (MBBCs) of BNEs, PA2, and PA-BNE against *S. aureus* biofilm represented as a heatmap and (**b**) bacterial viability (%) of *S. aureus* biofilm at 16% of BNE varying PA2 concentrations after treatment. The data shown are averages of triplicates with the error bars indicating standard deviations.

We next evaluated the antimicrobial activity of PA in the nanoemulsions against *S. aureus* (CD-35) and *B. subtilis* (FD6b) biofilm. The minimum biofilm bactericidal concentrations (MBBCs) were initially determined for each component using high-throughput screening. The PA2 alone was 4 mg/L where BNE was >16% (Figure 3a) against *S. aureus*. For *B. subtilis*, PA2 was presenting limited antibiofilm activity (>100 mg/L). Subsequently, we used PA-BNE with different levels of PA2 loading to assess their combined effects and best formulations for delivering PA to biofilm through checkerboard titrations. We observed that PA2 was active at 16% (*v/v*) concentrations of BNE, showing eradication at 32-fold lesser concentrations (0.125 mg/L) with BNE than PA2 alone (Figure 3b). For *B. subtilis*, a similar trend was observed, where PA-BNE (with 4 mg/L of PA2) showed eradication of biofilms after treatment (Figure S2). The additive effect was determined between PA2 and eugenol by fractional inhibitory concentration index (FICI = 0.531). Furthermore, we tested PA-BNE against MRSA biofilms with vancomycin as a control to evaluate the therapeutic potential of the system. Notably, the PA-BNE showed the most reduction in bacterial viability as comparable to the antibiotics.

### 2.4. Cytotoxicity of PA-BNE In Vitro Fibroblast Cell 

Lack of toxicity to mammalian cells of combined PA-BNEs was demonstrated by mammalian cell viability test against 3T3 fibroblast cells, which is an epithelial cell line that is damaged during bacterial biofilm infections at the wound site. PA-BNEs showed safety towards mammalian cells, maintaining cell viability levels above 90% [30]. These results were comparable to those observed for the drug alone, indicating the safety of the combined system through nanoemulsions. Across the concentration range tested, PA-BNEs consistently demonstrated no significant adverse effects on cell viability, indicating its potential for safe implementation in various applications.

## 3. Discussion

Small molecule antimicrobials (SMAs) present a new and effective approach to developing therapeutics, offering a wide range of possibilities for drug design to combat drug-resistant infections [31,32]. Pseudopyronines (PAs) are a class of α-pyrone natural products, synthesized by multiple bacterial species [31,33]. In this study, we screened antimicrobial activity of synthesized derivatives of PAs, varying the number of carbons from 3,4 (PA1), 6,7 (PA2), and 7,8 (PA3) on C6 and C3 positions respectively. We confirmed the longer alkyl chains, but particularly PA2 with 6,7 carbons and a ketone on the C3 alkyl side chain, exhibited the highest potency against Gram-positive (*S. aureus*, MRSA and *B. subtilis*) strains of bacteria (Table 1). However, the application of SMAs is hindered by their low solubility in water, posing challenges in complex biological settings such as biofilms [31].

Polymers with poly(oxanorbornenimide) (PONI) backbones have been used as carriers to deliver antimicrobials [33]. We previously reported functionalized PONI-polymers with guanidinium, maleimide and tetramethylene glycol monoethyl ether (PONI-GMT) to form nanoemulsions with essential oils by using amphiphilic properties of polymers [26]. Essential oils are promising antimicrobials, but their activity against biofilm is reduced due to their hydrophobicity, which limits their penetration through the biofilm matrix [34]. Therefore, essential oils using nanoemulsions featured efficient penetration resulting in enhanced antimicrobial activity against biofilm [26]. BNEs were also used for the delivery of additional antimicrobials, such as hydrophobic triclosan [28]. In the current study, we employed nanoemulsions for the delivery of PAs to eradicate bacterial biofilms, using efficient loading of PAs to amphiphilic BNEs. 

The BNEs used in our studies are composed of both hydrophobic and hydrophilic materials, enabling the incorporation of hydrophobic antimicrobials like PAs. Firstly, we tested the compatibility of PAs with essential oil by dissolving PAs into eugenol. Eugenol was chosen for its broad antimicrobial activity and ability to disrupt biofilms forming stable nanoemulsions with PONI-GMT polymers [26,35,36]. The solubility of PAs was tested and PA2 showed the highest solubility (100 mg/mL) among the PAs (Table 2). PA2 also demonstrated potent antimicrobial activity, suggesting that PA2 can be a promising candidate for co-delivery with eugenol in BNEs to enhance the accumulation of therapeutics and achieve synergistic bacterial killing against the biofilms. 

Using the stock solution of PA2-eugenol, we generated formulations of PA-BNEs using emulsification (Figure 2a). We demonstrated the successful fabrication of nanoemulsions loaded with chosen analogs PA2, with size ~290 nm (Figure 2b, Figure S1) with positive surface charge +10 mV in an aqueous solution (Figure 2c). The formulated PA-BNEs were tested against MRSA to determine minimal bactericidal concentrations (MBCs) with PA2 and BNE controls. The result indicated PA-BNE featured an additive effect by lowering MBCs of PA2 and BNE when they were treated individually. Next, we screened formulations by varying PA2 concentrations to determine their synergistic effect between PA2 and eugenol using a checkerboard assay to determine MBBC. We demonstrated PA-BNEs showed a 32-fold reduction up to 0.125 mg/L relative to PA2 alone (4 mg/L). For *B. subtilis*, the MBBC of PA-BNE was determined at 4 mg/L with BNE while PA2 alone showed now significant antibiofilm activity (>100 mg/L). These results suggest the activity of PA2 was enhanced through the nanoemulsions system by enhanced delivery of PA2 across the mature biofilms (Figure 3a and Figure S2). 

We assessed the antimicrobial activity of PA-loaded nanoemulsions against biofilms of *S. aureus* (CD-35), *B. subtilis* (FD6b), and MRSA (IDRL-6169). For *B. subtilis*, PA-BNE (containing 4 mg/L of PA2) effectively eliminated the biofilms. Furthermore, we tested the PA-BNE against MRSA biofilms, using vancomycin as a control to evaluate the therapeutic potential of the system (Figure S3). Notably, the PA-BNE exhibited a significant reduction in bacterial viability, comparable to the effects of the antibiotics.

Our findings demonstrate that PA-loaded nanoemulsions, specifically PA-BNE, effectively target and eradicate biofilms of *S. aureus*, *B. subtilis*, and MRSA. The combination of PA2 and eugenol within the nanoemulsion formulation shows enhanced antibiofilm activity, highlighting the therapeutic potential of this system.

**Figure 4:**
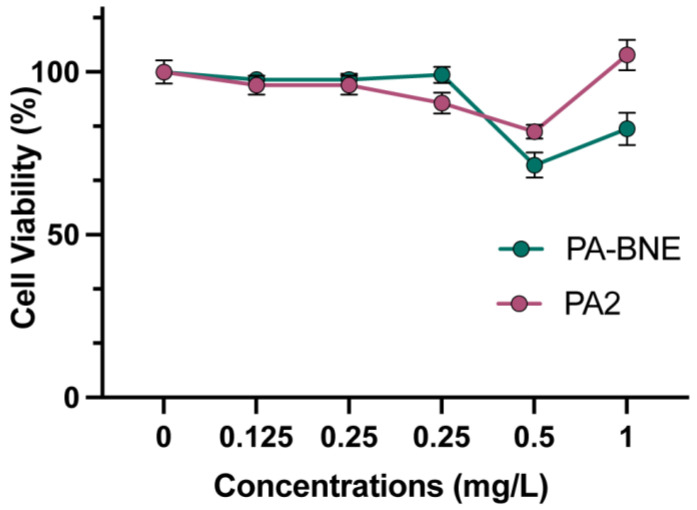
Cell viability of 3T3 fibroblast cells measured band quantified by Alamar blue assay. Treatment was performed for 3 h with materials. Values are expressed as mean ± standard deviation of ≥3 replicates.

Biofilm formation at infection sites can prolong the healing process regulated by fibroblast skin cells [37,38]. To evaluate the compatibility of PA-BNEs, we tested their cytotoxicity at concentrations used to eliminate established biofilms. In in vitro 3T3 fibroblast cells, PA-BNEs did not exhibit any significant toxicity towards fibroblast cells within the relevant concentration range (Figure 4). Overall, this result indicates that combination therapy of PA2 and BNEs has minimal toxicity towards mammalian cells. 

## 4. Materials and Methods

The following bacteria strains were used for this study: *S. aureus* (CD-35), MRSA (IDRL-6169), and *B. subtilis* (FD6b). Overnight cultures of bacteria were prepared by transferring the isolated colony from the agar plate to culture tubes with sterile media broth. The bacterial cultures were then incubated overnight at 37 °C with aeration and agitation (275 rpm) until they reached the desired growth phase. Isolates with code IDRL were from the Infectious Diseases Research Laboratory at Mayo Clinic (Rochester, MN, USA). CD were from the Cooley Dickenson (Northampton, MA, USA). NIH-3T3 cells (ATCC CRL-1658) were purchased from ATCC. Dulbecco’s Modified Eagle’s Medium (DMEM) (DMEM; ATCC 30-2002) and fetal bovine serum (SH3007103) (Thermo Fisher Scientific, Waltham, MA, USA) were used for cell culture. Invitrogen™ alamarBlue™ Cell Viability Reagent (DAL 1100) was purchase from Thermo Fisher Scientific (Waltham, MA, USA) and used following the manufacturer’s protocol.

### 4.1. Determination of Minimum Inhibitory Concentration (MIC) and Minimum Bactericidal Concentrations (MBCs)

The bacteria were grown in TSB medium under specific conditions. Once they reached the mid-log phase, the cultures were collected through centrifugation and washed with a sodium chloride solution. The concentration of the bacterial solution was determined by measuring the optical density at 600 nm. Dilutions of the bacterial solution were fabricated using M9 medium to achieve a concentration of 1 × 10^6^ cfu/mL. In a 96-well plate, 50 μL of these diluted bacteria in M9 was mixed with 50 μL of testing materials in M9, resulting in a final bacterial concentration of 5 × 10^5^ cfu/mL. The testing concentration was adjusted according to a standard protocol. Control groups, including a growth control with only a bacterial solution and a sterile control, were included in each experiment. The plate was then incubated for 16 h. The experiment was performed in triplicates, and at least two independent experiments were conducted on different days. The minimum inhibitory concentration (MIC) was determined as the lowest concentration of the testing chemical that prevented visible growth observed with the naked eye. Subsequently, the testing solutions (up to 4-fold MICs) exhibiting no visible growth were further diluted and enumerated on tryptic soy agar (TSA) plate. The MBC value was determined if it showed ~99% reduction in CFU/mL.

### 4.2. Preparation of PA-BNE 

PA-BNEs were created by emulsifying eugenol loaded with PA2 and DTDS into an aqueous solution of PONI-GMT. Solid PA2 was dissolved in eugenol at different concentrations up to 33 mg/mL, along with DTDS at a concentration of 3 wt %. A total of 3 μL of the oil mixture and 497 μL of the aqueous PONI-GMT solution were combined and emulsified using an amalgamator for 50 s. The resulting emulsions were left to rest overnight before being utilized in the experiment. 

### 4.3. Minimal Biofilm Bactericidal Concentration (MBBC) Determination 

The minimum biofilm bactericidal concentrations (MBBCs) of the BNEs, PA2, and PA-BNEs were determined following established protocols. Bacterial solutions were prepared from overnight cultures and diluted 1/50th using tryptic soy broth (TSB) and incubated at 275 rpm and 37 °C until they reached the mid-log phase. Next, 150 µL of the bacterial culture was added to each well of a 96-well microtiter plate with pegged lids and incubated at 37 °C and 50 rpm for 6 h. Subsequently, the pegged lids were rinsed by submerging them in 200 µL of PBS for 30 s, followed by transfer to a separate 96-well plate containing two-fold serial dilutions of the therapeutic agents prepared in 5% TSB in M9 media. The plate was then incubated at 37 °C for 24 h. Afterward, the biofilms on the lids were treated with PBS as described earlier and transferred to a new plate containing fresh media. This plate was further incubated at 37 °C to determine the MBBC. The MBBC of the antibiofilm agents was determined through visual inspection and confirmed using spectrophotometry by measuring the optical density at 600 nm (OD600).

### 4.4. Antibiofilm Study 

Bacterial seeding solutions were prepared in Tryptic Soy Broth (TSB) to achieve an optical density (OD) of 0.1. To a 96-well plate, 100 μL of the seeding solution was added. The plates were covered and incubated at room temperature under static conditions for 2 days to allow biofilm formation. After the incubation period, the biofilms were washed three times with phosphate-buffered saline (PBS) to remove any planktonic bacteria. PA-BNE fabricated in M9 media (5% *v/v* of TSB) were added to each well of the microplate. The microplate was then incubated at 37 °C under static conditions for 3 h. Following the incubation, the biofilms were washed with PBS three times, and the viability of the bacteria was assessed using an Alamar blue assay, following the manufacturer’s protocol. This assay provides a measure of bacterial viability.

### 4.5. Mammalian Cell Viability Assay 

The cytotoxicity of different components was evaluated using established protocols. To begin, 20,000 NIH 3T3 fibroblast cells (ATCC CRL-1658) were cultured in a 96-well plate using Dulbecco’s modified Eagle medium (DMEM, ATCC 30-2002) supplemented with 1% antibiotics and 10% bovine calf serum. The cells were incubated in a humidified atmosphere with 5% CO_2_ at 37 °C for 48 h. After incubation, the media was removed, and the cells were washed with phosphate-buffered saline (PBS) before incubation with the therapeutic agents. The PA2 or PA-BNE, solutions were prepared in media containing 10% serum and incubated with the cells in the 96-well plate for 3 h under humidified conditions at 37 °C. Alamar blue assays were performed following the manufacturer’s protocol from Invitrogen Bio-source to assess cell viability. The reduction in the Alamar blue agent resulted in red fluorescence, which was quantified using a Spectromax M5 microplate reader (Ex: 560 nm, Em: 590 nm). The percentage of cell viability was determined relative to the cells incubated with no materials, which were considered 100% viable controls. Each experiment was conducted in triplicate and repeated on two different days.

## 5. Conclusions

In summary, our study explored the use of small molecule antimicrobials (SMAs) and nanoemulsions for combating drug-resistant infections, particularly biofilms. Our studies found that longer alkyl chain derivatives of pseudopyronine analogs 2 (PA2) exhibited the highest potency against Gram-positive bacteria with MIC of 0.5 mg/L. By incorporating PAs into nanoemulsions, we enhanced their solubility and antimicrobial activity. The synergistic effect of PA2 and eugenol in the nanoemulsion system resulted in a significant reduction up to 32-fold less in biofilm eradication concentration. Importantly, our PA-loaded nanoemulsions demonstrated compatibility with mammalian cells. Overall, these findings indicate that SMA-carrier combinations offer a promising strategy to effectively target biofilms and combat multidrug-resistant infections.

## Figures and Tables

**Table 1:** In vitro antimicrobial activity of PAs.

Compound	MIC (mg/L)		
	*S. aureus*	MRSA	*B. subtilis*
PA1	500	>500	500
PA2	0.5	12.5	50
PA3	25	177	50

*S. aurues* (CD-35), MRSA (IDRL-6169), and *B. subtilis* (FD6b).

**Table 2:** Solubility test result of the PAs in eugenol.

	PA1	PA2	PA3
Drug concentrations in eugenol (mg/mL)	48	100	25

**Table 3:** Minimal bactericidal concentrations (MBCs) of PA2, BNE and PA-BNE against MRSA.

Materials	MBC
	MRSA (IDRL-6169)
PA2	12 mg/L
BNE	16% (*v/v*)
PA-BNE	8% (*v/v*), 4 mg/L

## Data Availability

Data are contained within the article.

## Supplementary Materials

The following supporting information can be downloaded at: https://www.mdpi.com/article/10.3390/antibiotics12081240/s1, Figure S1: Dynamic Light Scattering Histogram of PA-BNE as of Intensity (percent); Figure S2: Minimum biofilm bactericidal concentrations (MBBC) of BNEs, PA2, and PA-BNE against B. subtilis biofilms represented as a heatmap after overnight treatment. Figure S3. Bacterial viability of MRSA (IDRL-6169) biofilms after the treatment with materials and vancomycin control.
