# Supplementary material for: Integration of Antimicrobials and Delivery Systems: Synergistic Antibiofilm Activity with Biodegradable Nanoemulsions Incorporating Pseudopyronine Analogs
Source: Antibiotics (Basel). 2023 Jul 28;12(8):1240. doi: 10.3390/antibiotics12081240 (PMC10451319; doi:10.3390/antibiotics12081240)
Supplement: Supplementary file 1 [file antibiotics-12-01240-s001.zip › antibiotics-2500855-supplementary.pdf]

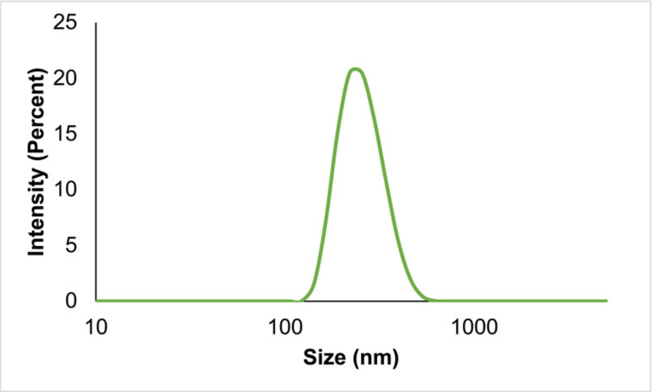

Figure S1. Dynamic light scattering histogram of PA-BNE as of Intensity (percent). The peak was observed ~255 nm

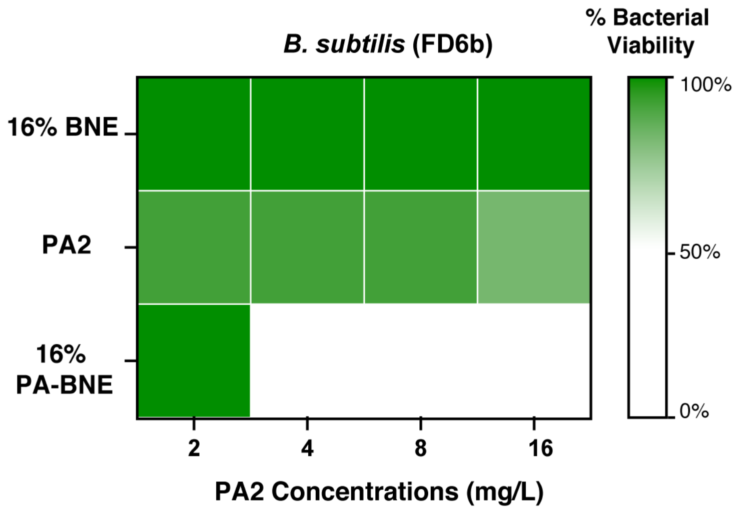

Figure S2. Minimum biofilm bactericidal concentrations (MBBC) of BNEs, PA2, and PA-BNE against *B. subtilis* biofilms represented as a heatmap after overnight treatment. The MBBC of PA2 alone was >100 mg/L.

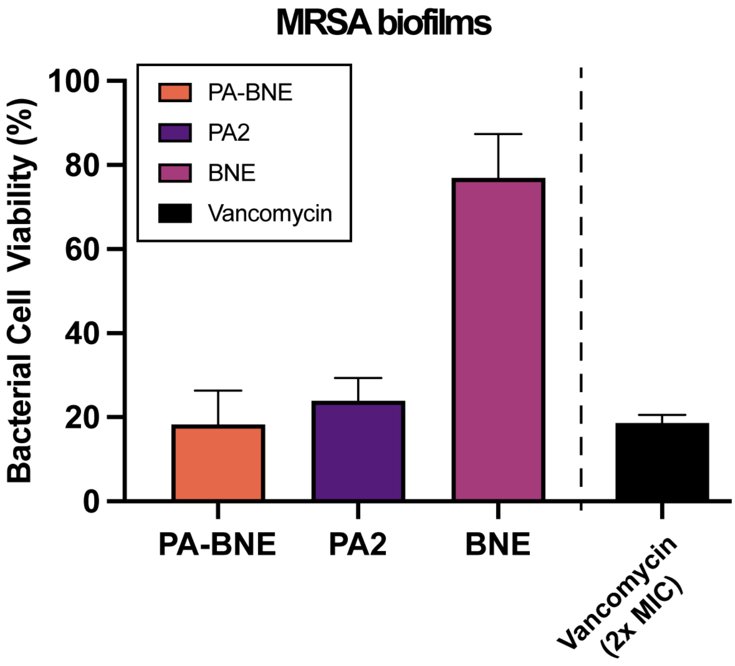

Figure S3. Bacterial viability of MRSA (IDRL-6169) biofilms after the treatment with materials and vancomycin control. The data shown are averages of replicates (n=5) with the error bars indicating standard deviations.PA-BNE (16%, 8 mg/L), PA2 (24 mg/L), BNE (16%) and 2x of MIC vancomycin (4 mg/L)
